# Supplementary material for: Impact of economic indicators on rice production: A machine learning approach in Sri Lanka
Source: PLoS One. 2024 Jun 21;19(6):e0303883. doi: 10.1371/journal.pone.0303883 (PMC11192421; doi:10.1371/journal.pone.0303883)
Supplement: Attachment — Submitted filename: Response_to_Reviewer_2_Comments.pdf [file pone.0303883.s001.pdf]

Response letter for manuscript PONE-D-24-05247

April 6, 2024

Reviwer 1 Comments

1 The novelty and contribution of the paper should be clearly stated.

Authors' responses:
Thank you for your suggestion. We have updated the introduction stating the novelty and contribution of this study. The updated content is highlighted for your reference.

Novelty:

This study presents a novel and comprehensive analysis of the multifaceted relationships between economic indicators and rice production in Sri Lanka, integrating machine learning approaches to uncover correlations and build prediction models, with findings directly applicable to the local agricultural landscape and potential implications for evidence-based policies and initiatives.

Contributions:

- Comprehensive Analysis: This research aims to provide a thorough investigation of the multifaceted relationships between economic indicators and rice production in Sri Lanka, offering a more comprehensive understanding of the factors at play.
- Integration of Machine Learning: By incorporating machine learning approaches, the study seeks to uncover correlations and build prediction models that can provide insights into the complex dynamics of economic forces and rice cultivation.
- Potential Policy Implications: The findings of this research could inform evidence-based policies and initiatives that promote a resilient future for Sri Lankan rice production, highlighting the practical applications of the study's insights for policymakers and agricultural stakeholders.

2 What is the contribution of this finding in addressing the challenges that were identified in the introduction?

Authors' responses:
Thank you for the question.

The findings of this study contribute significantly to addressing the challenges identified in the introduction by offering a comprehensive analysis of the complex relationships between economic indicators and rice production in Sri Lanka. By integrating machine learning approaches, the study provides a deeper understanding of these relationships, which can inform evidence-based policies and initiatives to promote a resilient future for Sri Lankan rice production. Additionally, the focus on the local context ensures that the findings are directly applicable to the specific challenges faced by the country's agricultural sector, bridging the gap in the literature and providing actionable insights for policymakers and agricultural stakeholders.

We have updated the introduction with this information. The updated content is highlighted for your reference.

3 What authors were motivated to conduct this type of study, and why did they select the Sri Lanka as a case study?

Authors' responses:
Thank you for the question.

The authors were motivated to conduct this study by the need for a deeper understanding of the complex relationships between economic indicators and rice production, particularly in the context of Sri Lanka. Political instability in recent years is also a motivating factor for conducting this study in Sri Lanka. Political instability can have significant implications for the economy, including the agricultural sector. By studying the relationship between economic indicators and rice production in Sri Lanka, the authors may aim to provide insights that can help mitigate the impact of political instability on rice cultivation and inform more stable and resilient agricultural policies. Additionally, the study seeks to understand how political factors interact with economic indicators to influence rice production, providing valuable insights for policymakers navigating the challenges of political uncertainty.

We updated the manuscript to answer your question. The updated content is highlighted for your reference.

4 Plenty of work has already proved the potential of various ML models across the world. What is different being using the applied models in this study?

Authors' responses:
Thank you for your question.

While previous work has demonstrated the potential of various machine learning (ML) models in agricultural research, including predicting crop yields and analyzing economic factors, this study differs in several key aspects.

This study stands out by offering a nuanced examination of the intricate relationships between economic indicators and rice production specifically tailored to the Sri Lankan con-

text. By focusing on Sri Lanka, the research addresses a critical gap in the literature, providing insights that are directly applicable to the unique challenges and dynamics of the country's agricultural sector. The study's comprehensive approach, which integrates multiple machine learning models such as Gaussian Process Regression (GPR), Support Vector Machine (SVM), Linear Regression, Ensemble bagging, and Decision Tree, offers a thorough analysis of the factors influencing rice production. Additionally, the study's potential to inform evidence-based policies and initiatives for sustainable rice cultivation in Sri Lanka underscores its practical relevance and contribution to agricultural research and policymaking.

We updated the manuscript with this information. The updated content is highlighted for your reference.

5 What were the used input parameters?

Authors' responses:
We have utilized the following inputs to build the system to predict rice production.

GDP ($B)
Inflation (%)
Manufacturing ($B)
Population
Growth Rate (%)
Imports ($B)
Arable Land
Military ($B)

This information is included in the Methodology section. We have highlighted the section for your reference.

6 Provide the range of statistical evaluating standards and support them by citing suitable references. The authors can cite these articles.

Authors' responses:

Thank you for the comment. We cited the suggested articles by enriching our manuscript with their information.

7 Likewise, what is the transferability of such results to other locations in terms of impact or usefulness?

Authors' responses:

Thank you for the comment.

The transferability of the study's results to other locations can be significant, particularly in regions facing similar challenges of political instability and where rice is a staple food. The findings can offer valuable insights into how economic indicators impact rice production, which can be applied to other countries experiencing political turmoil. Additionally, since

many countries rely on rice as a staple food, the study's results can be useful for policymakers and stakeholders globally, providing a framework for understanding the complex interplay between economic factors and rice cultivation. By considering the specific context of Sri Lanka's political instability and the widespread use of rice as a staple, the study's findings can serve as a blueprint for enhancing agricultural sustainability and economic resilience in other regions facing similar circumstances.

The updated content is highlighted for your reference.

8 Include all the figures and tables at their respective place in the text.

Authors' responses:

Thank you for the comment. We rechecked the manuscript and placed the tables and figures in their respective places.

9 Authors need to consult with a native English speaker for language improvement.

Authors' responses:

Thank you for the comment. We had a native English speaker review the manuscript and also utilized Grammarly to optimize the sentence further.

10 Reveal more practical debate in the discussion section and explain the usefulness and practical implications of this work.

Authors' responses:

Thank you for the comment.

The study's comprehensive analysis of the relationship between economic indicators and rice production in Sri Lanka offers valuable insights that can inform evidence-based policy making and agricultural strategies. By uncovering the complex interplay of factors influencing rice production, the study provides a nuanced understanding of the challenges and opportunities facing the agricultural sector in Sri Lanka.

One practical implication of the study is its potential to guide policymakers in developing strategies to enhance rice production and agricultural sustainability. By identifying key economic indicators that impact rice cultivation, policymakers can tailor interventions to address specific challenges and capitalize on opportunities for growth. For example, the study's findings suggest that focusing on boosting the manufacturing sector and maintaining price stability could positively influence rice production. Similarly, strategies to improve arable land quality and manage inflation could lead to more resilient agricultural systems.

Furthermore, the study's use of machine learning approaches adds a new dimension to its practical implications. By demonstrating the effectiveness of these techniques in predicting rice production outcomes, the study highlights the potential for integrating advanced technologies into agricultural decision-making. This could lead to more accurate and timely predictions, allowing farmers and policymakers to respond proactively to changing conditions.

We updated the manuscript with this information. The updated content is highlighted for your reference.

-

Note: The updated content is highlighted in the manuscript for your reference.
The authors appreciate all of the insightful comments from the editor. We worked hard to be responsive to them. Thank you for taking the time and energy to help us improve the paper.

Thank you.
